# Supplementary material for: Identification of photoperiod-induced specific miRNAs in the adrenal glands of Sunite sheep (Ovis aries)
Source: Front Vet Sci. 2022 Jul 22;9:888207. doi: 10.3389/fvets.2022.888207 (PMC9354845; doi:10.3389/fvets.2022.888207)
Supplement: Supplementary file 1 [file Data_Sheet_1.ZIP › Supplementary file/miRDeep2 file.zip!/miRDeep2 file/Know_Structure/SPLP42A1/oar-mir-134.pdf]

miRBase precursor : oar-mir-134
Total read count : 117
oar-miR-134-5p read count : 105
oar-miR-134-3p read count : 12
remaining reads : 0

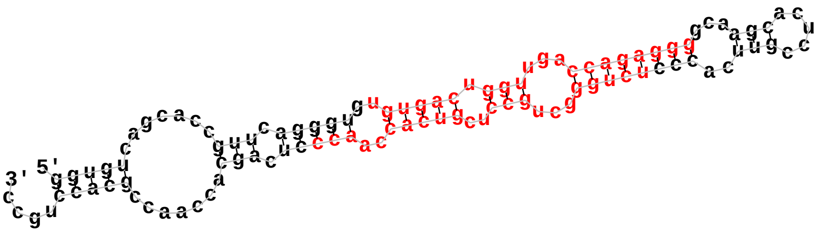


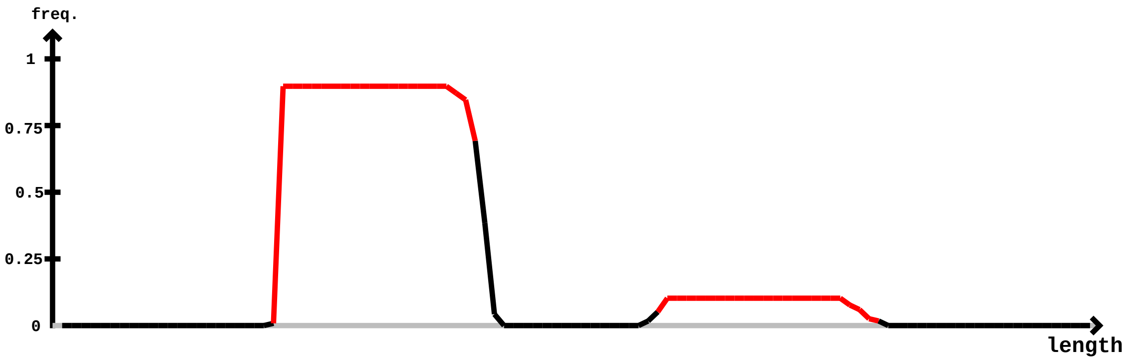


```
                                                                 oar-miR-134-3p
                       oar-miR-134-5p
5'- ggugucagcaccguucagggugugugacugguugaccagagggggcaagcacuccguucacccucugggcugccucgucaccaacccucagcaccaaccgcaccugcc -3'   exp
    (((((.......(((.(((((..(((((.(((...((((((((...(((.....)))..))))))))...)))..)))))..))))).))).......)))))....   reads   mm   sample
    .....................gugugacugguugaccaga...................................................................   1       0    seq
    ......................ugugacugguugaccaga...................................................................   2       0    seq
    ......................ugugacugguugaccagag..................................................................   3       0    seq
    ......................ugugacugguugaccagagg.................................................................   18      0    seq
    ......................ugugacugguugaccagaggg................................................................   36      0    seq
    ......................ugugacugguugaccagagggg...............................................................   40      0    seq
    ......................ugugacugguugaccagaggggc..............................................................   5       0    seq
    ............................................................ccucugggcugccucgucacc..........................   1       0    seq
    ............................................................ccucugggcugccucgucaccaa........................   1       0    seq
    .............................................................cucugggcugccucgucacc..........................   1       0    seq
    .............................................................cucugggcugccucgucacca.........................   1       0    seq
    .............................................................cucugggcugccucgucaccaa........................   2       0    seq
    ..............................................................ucugggcugccucgucacc..........................   1       0    seq
    ..............................................................ucugggcugccucgucacca.........................   1       0    seq
    ..............................................................ucugggcugccucgucaccaa........................   1       0    seq
    ..............................................................ucugggcugccucgucaccaac.......................   1       0    seq
    ..............................................................ucugggcugccucgucaccaacc......................   2       0    seq
```
